# *Berberis vulgaris* L. Root Extract as a Multi-Target Chemopreventive Agent against Colon Cancer Causing Apoptosis in Human Colon Adenocarcinoma Cell Lines

**DOI:** 10.3390/ijms25094786

**Published:** 2024-04-27

**Authors:** Anna Och, Marta Kinga Lemieszek, Marek Cieśla, Dariusz Jedrejek, Aleksandra Kozłowska, Sylwia Pawelec, Renata Nowak

**Affiliations:** 1 Department of Pharmaceutical Botany, Medical University of Lublin, 1 Chodźki St., 20-093 Lublin, Poland; anna.ochagn@wp.pl; 2 Department of Medical Biology, Institute of Rural Health, 20-090 Lublin, Poland; martalemieszek@gmail.com; 3 College of Medical Sciences, University of Rzeszow, 35-310 Rzeszow, Poland; mciesla@ur.edu.pl; 4 Department of Biochemistry and Crop Quality, Institute of Soil Science and Plant Cultivation—State Rsearch Institute, Czartoryskich 8 Street, 24-100 Puławy, Poland; djedrejek@iung.pulawy.pl (D.J.); spawelec@iung.pulawy.pl (S.P.); 5 Department of Radiotherapy, Medical University of Lublin, 13 Radziwiłłowska St., 20-080 Lublin, Poland; aleksandrakozlowska@umlub.pl

**Keywords:** *Berberis vulgaris* L., antioxidants, anti-inflammatory, antiproliferative, colon cancer, LS180, HT-29, inflammatory bowel disease, phytopharmacology

## Abstract

*Berberis vulgaris* L. (*Berberidaceae*) is a shrub that has been widely used in European folk medicine as an anti-inflammatory and antimicrobial agent. The purpose of our study was to elucidate the mechanisms of the chemopreventive action of the plant’s methanolic root extract (BVR) against colon cancer cells. Studies were conducted in human colon adenocarcinoma cell lines (LS180 and HT-29) and control colon epithelial CCD841 CoN cells. According to the MTT assay, after 48 h of cell exposure, the IC_50_ values were as follows: 4.3, 46.1, and 50.2 µg/mL for the LS180, HT-29, and CCD841 CoN cells, respectively, showing the greater sensitivity of the cancer cells to BVR. The Cell Death Detection ELISAPLUS kit demonstrated that BVR induced programmed cell death only against HT-29 cells. Nuclear double staining revealed the great proapoptotic BVR properties in HT-29 cells and subtle effect in LS180 cells. RT-qPCR with the relative quantification method showed significant changes in the expression of genes related to apoptosis in both the LS180 and HT-29 cells. The genes *BCL2L1* (126.86–421.43%), *BCL2L2* (240–286.02%), *CASP3* (177.19–247.83%), and *CASP9* (157.99–243.75%) had a significantly elevated expression, while BCL2 (25–52.03%) had a reduced expression compared to the untreated control. Furthermore, in a panel of antioxidant tests, BVR showed positive effects (63.93 ± 0.01, 122.92 ± 0.01, and 220.29 ± 0.02 mg Trolox equivalents (TE)/g in the DPPH•, ABTS•+, and ORAC assays, respectively). In the lipoxygenase (LOX) inhibition test, BVR revealed 62.60 ± 0.87% of enzyme inhibition. The chemical composition of BVR was determined using a UHPLC-UV-CAD-MS/MS analysis and confirmed the presence of several known alkaloids, including berberine, as well as other alkaloids and two derivatives of hydroxycinnamic acid (ferulic and sinapic acid hexosides). The results are very promising and encourage the use of BVR as a comprehensive chemopreventive agent (anti-inflammatory, antioxidant, and pro-apoptotic) in colorectal cancer, and were widely discussed alongside data from the literature.

## 1. Introduction

Colorectal cancer is a primary malignant tumor of the large intestine, originating in the mucosa of the epithelium of the large intestine [1,2]. According to the World Health Organisation, it is the most common cancer, along with breast, lung, rectum, and prostate cancers; in 2020, colon cancer was third in terms of occurrence (1.93 million cases) and second in terms of mortality (916,000 deaths) [3]. Lifestyle factors associated with an increased risk of colorectal cancer include diet, obesity and metabolic syndrome, physical inactivity, smoking, and moderate to heavy alcohol consumption [4]. However, non-modifiable risk factors in the development of the disease also exist. One of them is inflammatory bowel disease. Inflammatory bowel disease significantly increases the risk of developing colorectal cancer and is crucial in its pathogenesis; it may occur from the initial stage of tumorigenesis and facilitate the development of colorectal cancer. Inflammatory bowel disease, its spread, and its exacerbation are also closely related to oxidative stress, which leads to damage to gastrointestinal cells, including DNA damage, protein aggregation, and membrane dysfunction. Several genetic risk loci for inflammatory bowel disease related to oxidative stress exist, and according to the available research, oxidative stress is significantly involved in the development of colorectal cancer [5]. Another non-modifiable risk factor for colorectal cancer is the presence of polyps. Histological progression from polyps to cancer is the result of a series and/or accumulation of genetic changes. Over time, these mutations can cause a loss of function of the *TP53* gene, which is a master regulator of transcription and apoptosis, affecting a wide range of cellular functions and ultimately leading to carcinogenesis [4].

One of the most important directions of research in the fight against cancer diseases is chemoprevention. It involves the introduction of external factors, such as medications or supplements, to stop or delay the onset, progression, or recurrence of cancer. Single compounds or extracts from plants have been proven to interfere with a specific stage of the carcinogenic process. The US Food and Drug Administration (FDA) has approved several agents for clinical use in cancer prevention [6,7]. Currently, research attention is especially focused on intracellular signaling cascades as common molecular targets for various chemopreventive phytochemicals. In the case of well-known mechanisms in the development of given cancers, chemoprevention can be used at a specific stage of development and for a specific biochemical pathway. In patients at risk for colorectal cancer, the most important processes that require chemoprevention are oxidative stress, inflammation, and apoptosis disorders. Substances of plant origin are currently one of the most intensively researched groups of chemical substances in terms of their chemopreventive effects, and the most important direction in chemoprevention for colorectal cancer is the search for new antioxidant and anti-inflammatory substances with pro-apoptotic effects. 

*Berberis vulgaris* L. (*Berberidaceae*) is a medicinal plant of the genus *Berberis* L. with more than 3000 years of tradition in folk medicine—other roots of the genus *Berberis* are listed in the Chinese Pharmacopoeia under the name “Sankezhen” [8], and Ayurvedic medicine also uses the root and bark of *B.s vulgaris* L. The species was also widely used in traditional medicine in Europe due to its beneficial effects on cardiovascular and liver diseases [9]. Also, the anti-inflammatory effect of the raw material was traditionally appreciated [10]. Despite its well-established traditional use for various inflammatory conditions, scientific research reveals little about the anti-inflammatory activity of *B. vulgaris* root and focuses on the content of its secondary metabolites with anti-inflammatory activity rather than the assessment of the root extract itself [11,12]. The anti-inflammatory effect of *B. vulgaris* has been confirmed in research, but mainly in the range of the action of fruit extracts [13,14,15,16,17]. 

*B. vulgaris* L. is currently appreciated in the Middle East as a medicinal and culinary plant. However, in the Western world, it was forgotten after it was devastated for economic and agricultural reasons in the twentieth century. Due to the intensification of scientific reports on the health-promoting effects of berberine, which is currently being intensively studied, there is an increasing interest in plant raw materials in which it is present. *B. vulgaris* L. itself is currently becoming increasingly popular among scientists looking for new sources of chemopreventive and chemotherapeutic agents due to its anticancer effects [18,19,20,21,22], and some health benefits of the use of *Berberis vulgaris* L. have already been assessed clinically [23]. 

*B. vulgaris* is a typically alkaloid plant material and its root is the most pharmacologically active part of the plant. Its pharmacological effect is likely related to its alkaloid composition, which has been extensively studied for its anticancer properties, especially against colon cancer. A literature analysis of the activity of the metabolites present in *B. vulgaris* L., including those unidentified in this study, clearly shows that its anticancer activity against colorectal cancer should be one of the primary and most justified research directions concerning BVR. In this study, the chemopreventive and antiproliferative potential of BVR against colorectal cancer was assessed for the first time. The antioxidant, anti-lipoxygenase, and proapoptotic activity of BVR were evaluated against human colon cancer cell lines. Furthermore, the influence of BVR on the expression of pro-apoptotic genes was examined for the first time. Simultaneously, the chemical composition of BVR was determined to confirm and clarify the influence of the qualitative and quantitative composition of its metabolites on the biological activity of this extract. 

## 2. Results

### 2.1. Biological Activity of BVR

#### 2.1.1. Antiradical and Antilipooxigenase Activity of BVR

The study was carried out using three methods, each of which demonstrated the antioxidant character of BVR. The DPPH• analysis showed that BVR is characterized by scavenging ability at the level of 63.93 ± 0.006 mg/g, expressed as Trolox equivalents (mg Trolox/g of dry extract). The ORAC results revealed the oxygen radical absorbance capacity (ORAC) for BVR at a level of 220.29 ± 0.02 mg/g, expressed as Trolox equivalents (mg Trolox/g of dry extract). The ABTS•+ results showed that the antiradical capacity (ABTS•+) of BVR was at the level of 122.92 ± 0.01 mg/g, expressed as Trolox equivalents (mg Trolox/g of dry extract). To investigate the anti-inflammatory properties of BVR, an experiment was carried out with one of the enzymes involved in the development of inflammation—lipooxygenase (LOX). Therefore, the direct ability of BVR for LOX activity was tested. Table 1 shows the percentage of inhibition of LOX for concentrations of 1 mg of the dry extract/mL reaction mixture.

#### 2.1.2. Influence of BVR and Its Main Constituents on the Viability of CCD841 CoN, LS180, and HT-29 Cells Using the MTT Assay 

**Figure 1:**
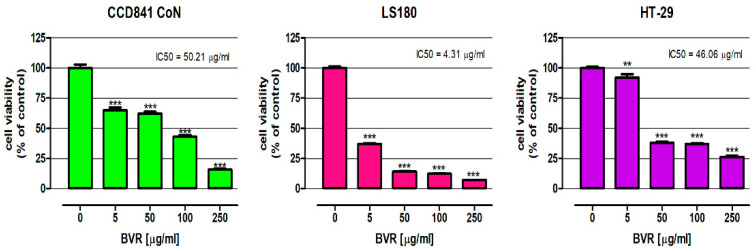
The effect of the BVR on the viability of human colon epithelial cell line CCD841 CoN and the human colon adenocarcinoma cell lines LS180 and HT-29. The results are presented as mean ± SEM (*n_min_* = 5). Data were analyzed using one-way ANOVA followed by post hoc Dunnett’s test: ** *p* < 0.01; *** *p* < 0.001 versus control.

The influence of BVR and its biologically active constituents, berberine, palmatine, and berbamine, on the viability of the human colon epithelial cell line CCD841 CoN and human colon adenocarcinoma cell lines LS180 and HT-29 was examined using an MTT assay. Studies were carried out after 48 h of treatment. As presented in Figure 1, BVR significantly decreased the metabolic activity of the normal and cancer cells and the observed effect was dose-dependent. Based on the results of the MTT test, the IC_50_ of the BVR values were determined for individual cell lines: CCD841 CoN = 50.21 µg/mL, LS180 = 4.31 µg/mL, and HT-29 = 46.06 µg/mL. Simultaneously, the IC_50_ doses of berberine, palmatine, and berbamine were calculated based on the results of the MTT assay and are shown in Table 2. 

**Figure 2:**
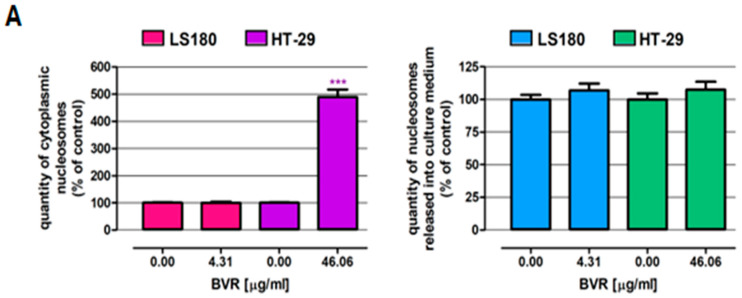

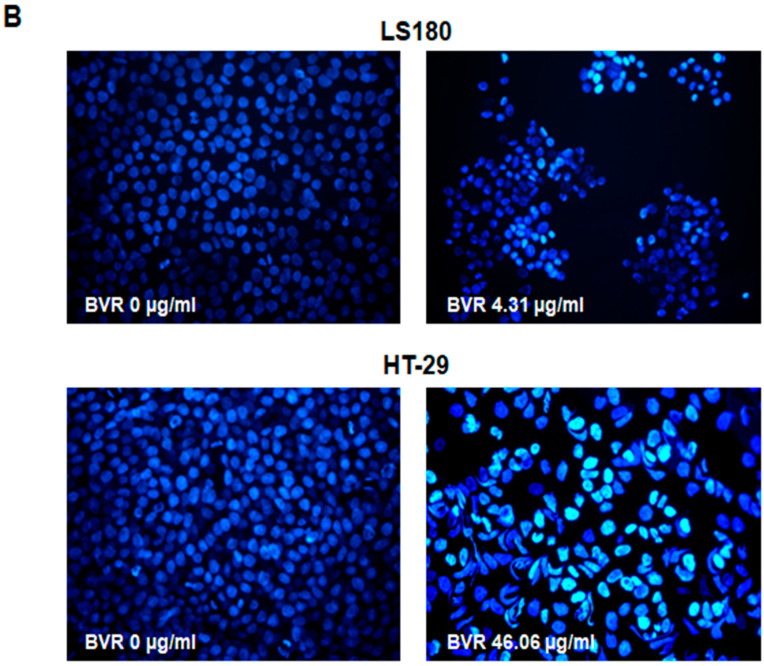
Pro-apoptotic effect of BVR in human colon adenocarcinoma cell lines LS180 and HT-29. Cancer cells were exposed to BVR at the IC_50_ level (4.31 and 46.06 µg/mL for LS180 and HT-29 cells, respectively). (**A**) Enrichment of nucleosomes in the cytoplasmic fraction (an apoptosis marker) or cell culture medium (a necrosis marker) determined by a cell-death detection ELISA. The results are presented as mean ± SEM (*n* = 6). Data were analyzed using Student’s *t*-test (control vs. treated): *** *p* < 0.001. (**B**) Nuclear double staining (Hoechst and propidium iodide) cancer cells treated with BVR. Representative pictures from fluorescence microscopy are presented; the magnification is 200×.

In the next step, the cell death induction properties of BVR were examined in the LS180 and HT-29 human colon adenocarcinoma cell lines using the commercial cell death detection ELISA kit. Furthermore, colon cancer cell death in response to BVR was observed under a fluorescence microscope after nuclear double staining. Studies were carried out after 48 h of cell incubation with BVR at IC_50_ values specific for the investigated cells (IC_50_ LS180 = 4.31 µg/mL; IC_50_ HT-29 = 46.06 µg/mL). As presented in Figure 2A, BVR at the indicated concentration induced programmed cell death only in the HT-29 cells, where the number of cytoplasmic nucleosomes reached 489.2% that of the control. The discovered proapoptotic features of BVR confirmed the collected micrographs (Figure 2B). The results of ELISA show that BVR at the tested concentration was unable to induce apoptosis or necrosis in the LS180 cells (Figure 2A), nevertheless, differential staining revealed the single apoptotic cells in the investigated cell line treated with BVR (Figure 2B).

#### 2.1.3. PCR Analysis

The analysis of gene expression considered five genes associated with apoptosis—BCL2 (B-cell CLL/lymphoma 2), BCL2L1 (*BCL2 Like 1)*, BCL2L2 (BCL2 Like 2), CASP3 (caspase 3), and CASP9 (caspase 9), and was carried out in human colon cancer cells exposed to the IC_50_ dose of BVR. 

**Figure 3:**
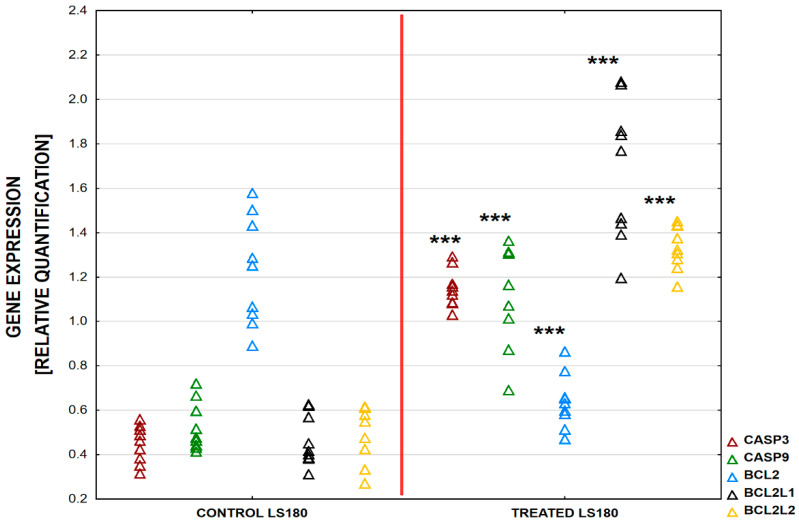
Differences in relative expression of five apoptotic genes (CASP3, CASP9, BCL2, BCL2L1, and BCL2L2) between LS180 cells treated with BVR and controls. The results are presented as raw data (*n* = 9). Abbreviations: *** *p*-value < 0.001.

**Figure 4:**
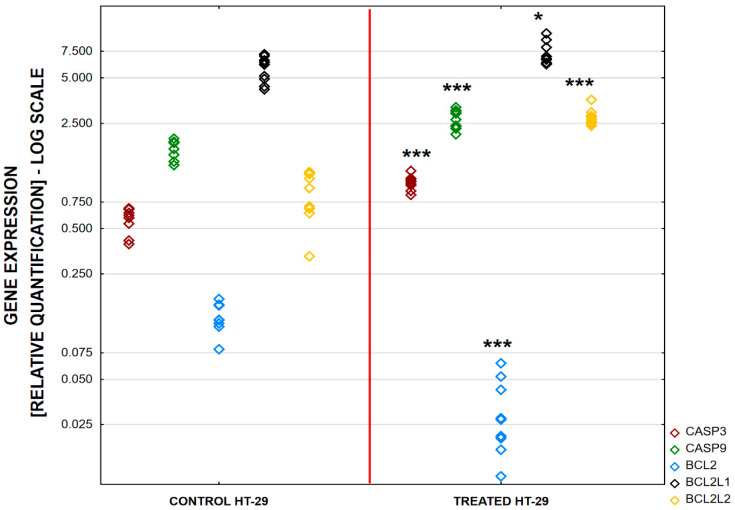
Differences in relative expression of five apoptotic genes (*CASP3*, *CASP9*, *BCL2*, *BCL2L1*, and *BCL2L2*) between HT-29 cells treated with BVR and controls. The results are presented as raw data(*n* = 9). Abbreviations: * *p* < 0.05 and *** *p* < 0.001.

An increased expression level of genes involved in apoptosis was observed in the LS180 cells. The highest increase was determined for *BCL2L1* (421.43%, *p* < 0.001) and the expression of *BCL2L2* was also elevated (240%, *p* < 0.001). An increased expression of genes encoding caspases was observed: *CASP3* (247.83%, *p* < 0.001) and *CASP9* (243.75%, *p* < 0.001). The expression of *BCL2* decreased to 52.03%, *p* < 0.0001(Figure 3) compared to the untreated controls. An increased expression level of genes involved in apoptosis was also observed in the HT-29 cells. The expression of *BCL2L1* was enhanced, but only at a level of 126.86%, *p* = 0.016. The expression of *BCL2L2* was also elevated to the highest level of 286.02%, *p* < 0.001. An increase in the expression of genes that encode caspases, *CASP3* (177.19%, *p* < 0.0001) and *CASP9* (157.99%, *p* < 0.0001), was also observed, but at a lower level than that recorded in the LS180 cells. In the HT-29 cells, *BCL2* expression also decreased to 25% in comparison to untreated samples, *p* < 0.001 (Figure 4). The relative gene expression is shown in Table 3. 

### 2.2. Phytochemical Profiling and Quantification of Major Specialized Metabolites 

**Figure 5:**
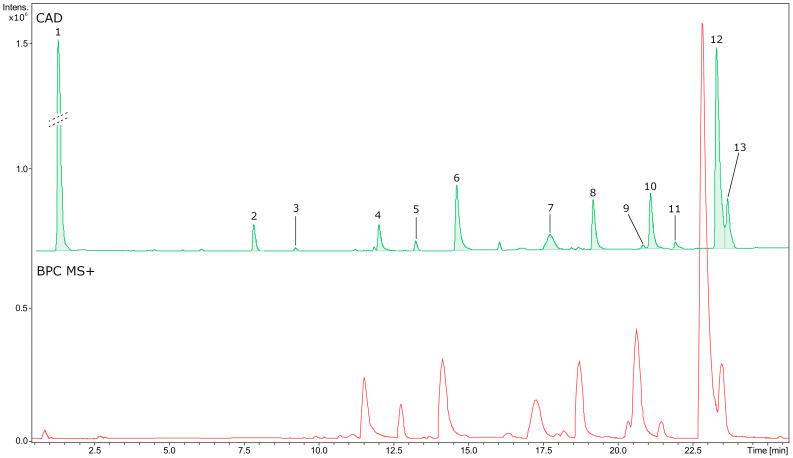
UHPLC-CAD-QTOF-MS analysis chromatograms of BVR. The peak numbers correspond to Table 3.

**Figure 6:**
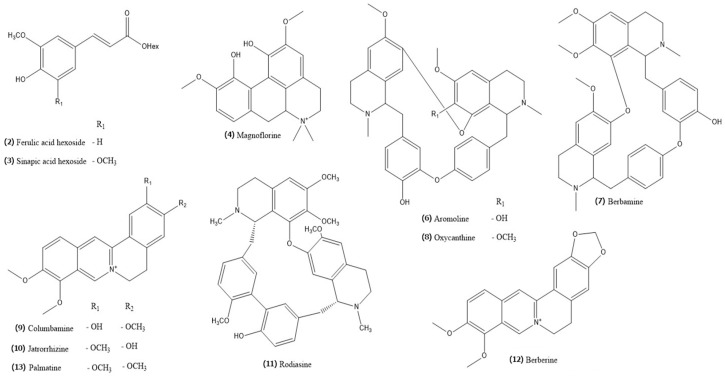
Chemical structures of metabolites determined in BVR.

The estimated phytochemical profile of the methanolic extract of the root of *B. vulgaris* (BVR) is presented in Figure 5 and a description of the detected metabolites can be found in Table 4. Moreover, the chemical structure of the identified compounds is shown in Figure 6. The main components determined in this study were alkaloids with a total content of 131.89 mg equivalent to berberine per 1 g of dry extract [mg BE/g BVR], specifying the main metabolite as berberine (70.27 mg/g BVR). 

## 3. Discussion

In this study, we evaluated the chemopreventive activity of BVR, and the antioxidant effect of the extract obtained from the root of *B. vulgaris* L. was the first step of this evaluation. There is little available in the literature on the antioxidant activity of *B. vulgaris* L., and, so far, research in this area has focused on fruits or other parts of this species [24]. Gorizpa et al. 2022 determined the antioxidant activity in the root extract of *B. vulgaris* subsp. asperma at the IC_50_ level of 89.70 ± 0.92 μg/mL and the values for the root extract of *B. vulgaris* subsp. orientalis were at the level of 167.24 ± 1.65 μg/mL [25]. El Khalki et al. 2018 reported an EC50 value of 69.65 μg/mL for the root ethanolic extract and 77.75 μg/mL for the root acetone extract [22]. Based on the chromatographical results obtained, it can be concluded that this antioxidant potential is caused by alkaloids with established antioxidant activity, which were determined in this study in BVR: berberine [26], palmatine [27], jatrorrhizine [28], and magnoflorine [29]. However, it is probable that there are other metabolites with antioxidant activity that have not yet been identified in BVR, which requires further research.

In addition to antioxidant activity, effective chemopreventive agents should also have anti-inflammatory activity. In traditional Iranian medicine, *B. vulgaris* is a known anti-inflammatory agent. Traditional European medicine has also used these properties in the past centuries to treat inflammation, especially diseases of the digestive system. Recent studies have shown that the main mechanisms of the anti-inflammatory effects of *B. vulgaris* are due to the content of berberine and include changes in the cellular immune response to Th2, Treg induction, the inhibition of inflammatory cytokines (IL-1, TNF, and IFN-γ), and the stimulation of IL-4 and IL-10 [30]. However, the anti-inflammatory effects of individual parts of the plant have not been studied so far. In this study, the anti-lipoxygenase activity of BVR was established at the level of 62 ± 0.87%. It is higher than the determined activity of the fruit (inhibition 45.24 ± 2.45%) but lower than the activity of the stem (83.57 ± 0.13%) and leaves (79.78 ± 2.19%) [24]. Currently, certain alkaloids marked in BVR are known to have anti-inflammatory properties and are probably responsible for its anti-inflammatory activity. The alkaloid with the best documented anti-inflammatory effect is berberine [31], which was determined in our study at a level of 70.27 ± 0.48 mg/g BVR. New reports also mention the anti-inflammatory activity of palmatine [28] and magnoflorine [29,32], which were determined in our study. The available data indicate the need for more detailed information on the composition of root extracts from *B. vulgaris*. 

The next step of the evaluation was the assessment of the cytotoxic activity of BVR towards colon cancer cells. As presented in Figure 2, BVR significantly decreased the metabolic activity of the normal and cancer colon cells, and the observed effect was dose-dependent. The most significant changes were observed in the LS180 cells, where BVR in the concentration range (5–250 µg/mL) caused decreases in cell viability of 62.76 and 92.90%, respectively. Surprisingly, BVR at the lowest concentration tested (5 µg/mL) reduced the viability of CCD841 CoN by 35.02%, while the metabolic activity of HT-29 was lowered by 7.10%. Nevertheless, in the remaining concentration range, the cancer cells were more sensitive to the effect of the extract than the normal cells.

Based on the results of the MTT test, the BVR IC_50_ values were determined for individual cell lines. CCD841 CoN = 50.21 µg/mL, LS180 = 4.31 µg/mL, and HT-29 = 46.06 µg/mL. A comparison of these IC_50_ values revealed a greater sensitivity of colon cancer cells than colon epithelial cells to BVR. Furthermore, differences in the sensitivity of the colon cancer cells to the proapoptotic effect of BVR were also observed. Simultaneously, the IC_50_ doses of berberine, palmatine, and berbamine against the investigated human colon cell lines were determined based on the results of the MTT assay and the data are shown in Table 3. A comparison of IC_50_ doses revealed that BVR was significantly more cytotoxic to the colon epithelial CCD841 CoN cells than berberine and palmatine. In contrast, it turned out to be the most toxic to normal cells. Significantly lower doses of both the extract and alkaloids reduced the metabolic activity of the colon cancer cell lines. In the case of the LS180 l cells, berbamine showed the relatively lowest cytotoxicity (IC_50_= 37.62 µg/mL; 61.8 µM) and berberine the highest (IC_50_ = 0.45 µg/mL; 1.33 µM). At the same time, berbamine turned out to be the most cytotoxic towards the HT-29 cells with an IC_50_ of 8.77 µg/mL (14.4 µM). There are not many studies that have evaluated the cytotoxicity of BVR; most research concerns single compounds present in the root of *B. vulgaris*. Abd El-Wahab et al. 2013 showed that root ethanolic extract from *B. vulgaris* inhibited the growth of breast, liver, and colon cancer cell lines (MCF-7, HepG2, and Caco-2, respectively) in a time- and dose-dependent manner. They also compared the effects of the extract and berberine on the proliferation of these cell lines. They described that the ethanolic extract of *B. vulgaris* was cytotoxic at concentrations ranging from 1 μg/mL up to 100 μg/mL [19]. El Khalki et al. 2018 showed that the ethanolic extract of *B. vulgaris* root bark is cytotoxic towards human breast adenocarcinoma cell lines (MCF-7) with an IC_50_ value of 3.54 μg/mL and no cytotoxicity to normal human peripheral blood mononuclear cells [22]. 

In regard to the presence of individual compounds in the extract, the main component determined in this study was berberine (70.27 mg/gBVR). Other metabolites determined in BVR were jatrorrhizine, magnoflorine palmatine, aromoline, columbamine, berbamine, and oxycanthine. Our results confirm the literature data [28,33,34]. Furthemore, berberrubine, berlamibine, lambertine, acanthine, and bargustanine were also recorded in the root of *B. vulgaris* L., but the data are mainly qualitative [10]. However, due to the variation in plant material caused, for example, by soil and climatic factors, there are also publications with slightly different levels of metabolites, as well as varied phytochemical profiles. Furthermore, two glycosidic derivatives of hydroxycinnamic acid (ferulic and sinapic) were identified and quantified in the BVR (in total, about 8 FAE mg/g of BVR). 

Among the alkaloids determined in this study, berberine is currently the widest investigated compound from the extract. It was described to be cytotoxic to the HT-29 cells after 48 h of exposure at a dose of 52.37 ± 3.45 μM and these cells were much more sensitive than the other cell lines tested: Tca8113—218.52 μM; CNE2—249.18 μM; MCF-7—272.15 μM; and HeLa—245.18 μM [35]. In the study of haematopoietic cell lines, berberine was cytotoxic in the range from 80.00 μM for CCRF/CEM cells to 250 μM for HL-60/MX2 cells [36]. Anticancer activity against several different cancer lines, including the HT-29 cells examined in this study, was also proven for the alkaloid palmatine, the presence of which has been reported in the investigated extract [37]. In addition, the berbamine determined in the investigated root extract exerts an anticancer effect by the induction of apoptosis. The results of Mou et al.’s 2019 research conducted in normal and cancer colon cells revealed the following IC_50_ values: HT-29 = 14 µM and IC_50_ CCD18 Co = 50 µM, which indicates a lower toxicity of berbamine against normal cells. Moreover, they described nuclear fragmentation after berbamine exposure, which indicates apoptosis induction in HT-29 cells after exposure to berbamine [38]. The anticancer activity of jatrorrhizine, which occurs in the root, stem, and bark of *B. vulgaris* L., has also been described. Zhang et al. 2014 proved that jatrorrhizine in the dose range of 100–500 µg/mL inhibited the cell growth and induced the apoptosis of HepG2 human hepatoma cells in a time- and concentration-dependent manner [39]. The available scientific data clearly suggested that the alkaloids contained in the BVR may influence the extract’s cytotoxicity Nevertheless, it seems that the final beneficial effect may be influenced by the yet undetermined secondary metabolites of the root. Thus, there is a great need to continue research on the content and influence of other chemical groups on the cytotoxic and pro-apoptotic activity of the root of *B. vulgaris* L., including, for example, tannins or terpene derivatives.

The qPCR analysis of the influence of BVR on the apoptotic gene expression in colon cancer cells is also consistent with that given in the literature regarding the individual alkaloids determined in BVR. They may be individually responsible for influencing the expression of the examined genes. However, next to alkaloids, other yet unidentified secondary metabolites may play role.

Currently, the best described compound of BVR also in the field of pro-apoptotic activity is berberine. The described induction of several biochemical events, that is, a reduction in the mitochondrial membrane potential, the release of cytochrome c, Bcl2 family proteins, and the activation of caspases or degradation of PARP after exposure to berberine, confirms the pro-apoptotic abilities of berberine. It is described that berberine induces apoptosis in cancer cells, mainly by upregulating pro-apoptotic genes and downregulating anti-apoptotic genes [36,40,41]. The pro-apoptotic effect of berberine has been demonstrated in colon in HCT-116 cells, where caspase 3-dependent apoptosis was demonstrated [42], while in the case of other colon cancer SW480 cells, apoptosis involving caspases 3 and 9 was proven [43]. Dai et al. 2019 described that long non-coding RNA cancer susceptibility candidate 2 (lncRNA CASC2) mediates the berberine-induced pro-apoptotic effect in colorectal cancer HT-29 cells by inhibiting Bcl-2 expression at the post-transcriptional level. Caspases 3 and 9 are also targets regulated by CASC2-regulated lncRNA that are related to berberine-induced apoptosis [44,45]. 

As mentioned above, the anticancer activity of jatrorrhizine, which occurs in the root, stem, and bark of *B. vulgaris*, has been also described. Sun et al. (2019) revealed a reduction in the levels of protein Bcl-2, procaspase-3, procaspase-8, procaspase-9, and PARP and an increase in the level of pro-apoptotic proteins BAX after exposure to jatrorrhizine in the MDA-MB-231, MCF-7, and 4T1 cell lines [46]. Wang et al. (2019) examined the effect of jatrorrhizine treatment on HCT-116 and HT-29 cells and also reported the down-regulation of procaspase-9 in HT-29 cells but an increase in HCT-116 cells. Further examination showed a slight reduction in procaspase-8 levels in the HT-29 cells and no significant changes in the HCT-116 cells. A slight reduction in the procaspase-3 level was observed in the HCT-116 cells without significant changes in the HT-29 cells [47]. The results indicated that the mechanism underlying its anticancer effect is the induction of apoptosis through caspase, including through the induction of ROS depletion [48], but the caspase-independent mechanism was also described [47]. The effect of jatrorrhizine on proteins in the Wnt signalling pathway has also been described, which are important regulators of cell proliferation and differentiation, whose signalling pathway is closely related to proteins that initiate apoptosis [49], gene transcription, and cell adhesion [50]. The metastasis-inhibiting activity of jatrorrhizine has also been shown through its influence on N-cadherin and E-cadherin in human HCT-116 and HT-29 colon cells [47]. Another alkaloid determined in the BVR with anticancer activity is palmatine. Using the example of a human skin epithelial cancer cell line A431, palmatine was shown to induce apoptosis depending on concentration and exposure time through severe DNA damage and inhibition of the activity of the Bcl-2 protein [51,52]. Wu et al. 2016 showed, also in HT-29 cells, that this compound induces early and late apoptosis, acting photodynamically [53]. Furthermore, Inbaraj et al. and Hirakawa et al. reported that palmatine can bind to DNA and destroy DNA through photooxidation, and then kill human keratinocyte line HaCaT [54,55]. Furthermore, palmatine inhibited the proliferation of human colorectal cancer cell lines by reducing the expression of the inflammatory cytokines IL-1a, IL1-b, and IL-8, granulocyte colony-stimulating factor, and granulocyte-macrophage colony-stimulating factor [56]. The columbamine also identified in the extract inhibited the proliferation, migration, and invasion of the colon cancer cells line HCT-116 and increased the rate of their apoptosis. Data on the mechanism of apoptosis in the case of this compound indicate that both signal transduction and the expression of key factors of the Wnt/β-catenin signaling pathway are suppressed in a dose-dependent manner [57]. Berbamine also identified in the BVR extract has demonstrated pro-apoptotic activity. According to Mou et al. 2019, berbamine activated caspase-3 and 9 and increased the Bax/Bcl-2 ratio in the colon cancer cell line HT-29. Additionally, it triggered the development of autophagic vesicles in HT-29 cells, which was concomitant with an increase in the protein levels of LC3B-I, ATG-5, ATG-12, and Beclin-1. Furthermore, in a wound healing assay, this compound decreased the migration potential of the mentioned cancer cells and also blocked their MEK/ERK signaling pathway [38]. 

It has also been shown that magnoflorine exerts anticancer effects in gastric cancer cells by regulating autophagic cell death, apoptosis, and cell cycle arrest in the S/G2 phase. Furthermore, magnoflorine inhibited AKT and activated JNK signaling, ROS-accumulation-dependent pathways that are associated with autophagy, apoptosis, and cell cycle arrest in various cancer cells [58]. It has been shown that magnoflorine increases the expression of caspase 3, responsible for apoptosis induction [29]. Some anticancer properties of other plant extracts containing magnoflorine have been also demonstrated, e.g., the inhibition of the expression of vascular endothelial growth factor (VEGF), the expression and stimulation of the angiogenesis process in hepatocellular carcinoma by the aqueous extract of the *Coptidis rhizome* [39], and the inhibition of the development of cervical cancer cells of the HeLa line, hepatocellular carcinoma cells of HepG2, and the brain tumor line U251 by the methanolic extract of *Magnolia grandiflora* leaves [59]; also, *Ziziphus jujuba* fruit extract, containing magnoflorine, shows a cytotoxic effect by inhibiting the proliferation of human breast cancer cell line MCF-7, human liver cancer cell line HepG2, and human colorectal adenocarcinoma cell line HT-29 [60]. Despite very intensive research on the anticancer activity of metabolites isolated from *B. vulgaris* L., there is still very little research on the activity of extracts from this plant. By analyzing the activity of other metabolites, also unidentified in this study, it is possible to outline research directions for this raw material. Anticancer activity against colorectal cancer seems to be one of the main and most justified research directions, as shown in Table 5.

Among the alkaloids determined in this study, oxycanthin was previously presented as a compound with no impact on the viability of different cancer cell lines. The literature data mentioned above indicate that alkaloids whose presence in BVR was confirmed in this study are largely responsible for the pro-apoptotic effects of *B. vulgaris* L., and the results of the biological activity of BVR are strongly supported by the literature data on its anticancer activity.

At the same time, the quantitative contents of the compounds determined in this study allow us to conclude that, when used in the extract, they have a more beneficial effect than when used separately at higher concentrations. This shows the advantage of a potential plant drug and the possibility of lowering the drug dose, which is one of the goals of phytopharmacology.

Furthermore, recent studies also show that, in addition to apoptosis, the isoquinoline alkaloids determined in BVR are promising in targeting angiogenesis and metastasis in colon cancer [49,53], which further broadens the prospects for the use of BVR as a new phytopharmaceutical drug. Subsequently, berberine and palmatine are also proven radiosensitizing agents, which also expands the possibilities of BVR being developed as a new phytopharmaceutical anticancer drug or adjuvant in cancer therapy.

## 4. Materials and Methods

### 4.1. Plant Material and Preparation of Extract

The root of *B. vulgaris* L. was obtained from the Botanical Garden of the Maria Curie-Skłodowska University of Lublin in September 2020 (the voucher specimen AO2020091 is deposited at the Department of Pharmaceutical Botany). The material was washed and dried in the shade with ventilation. Subsequently, the roots were ground in an electric mill to a homogeneous size and sieved through a 0.5 mm sieve. The powdered plant material was vacuum packed and stored at −30 °C until extraction.

In total, 2 g of powdered root was extracted with the Dionex ASE 150 accelerated solvent extractor (Sunnyvale, CA, USA) using 100% MeOH (*v*/*v*) at 80 °C and 1500 psi (solvent pressure), conducting three repeated extraction cycles. Subsequently, the BVRroot extract was evaporated to dryness under reduced pressure and lyophilized in a FreeZone 1 apparatus (Labconco, Kansas City, KS, USA). The estimated extraction efficiency was 10.29% [102]. 

For the phytochemical analyses, the freeze-dried extract was re-dissolved in 80% MeOH using an ultrasonic bath and stored at −20 °C until tested. 

For the bioassay tests, a 50 mg/mL stock solution was prepared with DMSO and it was stored at −20 ° C until tested. Working solutions of BVR were prepared prior to analysis by dilution with culture medium; the final concentration of DMSO in the test samples was <0.05% (*v*/*v*) and its effects were determined in the experiments.

### 4.2. Chemicals and Reagents

A 2,2-diphenyl-1-picrylhydrazyl (DPPH•), 2,20-azino-bis (3-ethylbenzothiazoline-6-sulfonic acid) (ABTS•+), Folin–Ciocalteu reagent, 2,20-azobis (2-methylpropionamide) dihydrochloride (AAPH), Trolox, acetonitrile and formic acid (both MS grade), berberine (95% purity), and ferulic acid were purchased from Sigma–Aldrich (Stenheim, Germany). Ascorbic acid was purchased from Stanlab (Lublin, Poland), and analytical-grade methanol and aluminium chloride hexahydrate were purchased from POCH (Gliwice, Poland). Ultrapure water was prepared with a Milli-Q purification system (Millipore, Burlington, MA, USA).

The SuperScript Vilo cDNA Synthesis kit was used according to the manufacturer’s recommendations. cDNA was stored at −20 °C until subsequent analysis. PCR reactions were performed using SG onTaq qPCR Master Mix (Eurx, Gdańsk, Poland) on the QuantStudio 5 Real-Time PCR System (Applied Biosystems, Waltham, MA, USA). 

### 4.3. Antiradical Activity Analyses 

#### 4.3.1. Determination of Antiradical Potential with the DPPH• Assay 

The assay was carried out according to Brand-Williams et al. [103], with some modifications [104]. The absorbance was measured after 60 min at 517 nm using an Infinite Pro 200F microplate reader (Tecan Group, Männedorf, Switzerland). The results are expressed as milligrams of Trolox equivalents per gram of dry extract [mg TE/g]. 

#### 4.3.2. Determination of Antiradical Capacity with the ABTS•+ Assay 

The assay was carried out according to Pellegrini et al. [105], with some modifications [106]. The absorbance was measured at 734 nm after 6 min of incubation. The level (%) was calculated as follows: [(Abs_control_ − Abs_sample_)/Abs_control_] × 100. The results are expressed as milligrams of Trolox equivalents per gram of dry extract [mg TE/g].

#### 4.3.3. Oxygen Radical Absorbance Capacity (ORAC) Assay 

The determination of the oxygen radical absorbance capacity (ORAC) was carried out according to a method developed by Huang et al. (2002) [107], with some modifications [104]. The activity of the sample is expressed as milligrams of Trolox equivalents per gram of extract [mg TE/g]. 

### 4.4. Lipoxygenase (LOX) Inhibitor Screening Assay 

The anti-lipoxygenase activity of BVR was determined according to Baraniak and Szymanowska [108]. The absorbance at 234 nm was measured immediately. The absorbance inhibition (%) was calculated as follows: [(Abs_control_ − Abs_sample_)/ Abs_control_] × 100.

### 4.5. Cell Cultures

The human colon epithelial cell line CCD841 CoN was purchased from the American Type Culture Collection (ATCC, Menassas, VA, USA). The human colon adenocarcinoma cell lines LS180 and HT-29 were obtained from the European Collection of Cell Cultures (ECACC, Centre for Applied Microbiology and Research, Salisbury, UK). The CCD841 CoN cells were grown in Dulbecco’s modified Eagle’s Medium (DMEM). Both the LS180 and HT-29 cells were grown in Dulbecco’s modified eagle medium/nutrient mix F-12 Ham (DMEM/F12). All cell culture mediums were supplemented with 10% foetal bovine serum (FBS), penicillin (100 U/mL), and streptomycin (100 μg/mL). The cells were maintained in a humidified atmosphere of 95% air and 5% CO_2_ at 37 °C.

### 4.6. Cell Viability Assessment—MTT Assay

The cells were seeded on 96-well microplates at a density of 5 × 10^4^ cells/mL. The next day, the culture medium was removed and the cells were exposed to BVR at concentrations of 5, 50, 100, and 250 μg/mL. After 48 h of incubation, under standard conditions (5% CO_2_, 37 °C), the MTT solution (5 mg/mL in PBS) was added to the cells for 3 h. The resulting crystals were solubilized overnight in SDS buffer at pH 7.4 (10% SDS in 0.01 N HCl), and the product was quantified spectrophotometrically by measuring the absorbance at a 570 nm wavelength using the microplate reader (BioTek ELx800, Highland Park, Winooski, VT, USA). The results are presented as a percentage of the metabolic activity of the cells treated with the extract versus the cells grown in the control medium (indicated as 100%).

### 4.7. Cell Death Assessment—ELISA

The cells were seeded on 96-well microplates at a density of 5 × 10^4^ cells/mL. The next day, the culture medium was removed and the cells were exposed to BVR at concentrations equal to the IC_50_ values calculated on the basis of the results of the MTT assay performed on the cell lines. After 48 h of treatment, cell death (both apoptosis and necrosis) was assessed using the Cell Death Detection ELISAPLUS kit (Roche Diagnostics GmbH, Mannheim, Germany) according to the manufacturer’s instructions. Assessments were conducted in cytoplasmic fractions, as well as in the cell medium collected from above the cell cultures, leading to determining the amount of nucleosomes in the apoptotic and necrotic cells, respectively. The absorbance was measured at a wavelength of 405 nm using a BioTek ELx800 microplate reader. The results are presented as the percentage of cells that underwent apoptosis/necrosis in response to the investigated compound versus the number of dying cells presented in the untreated control cells (indicated as 100%).

### 4.8. Cell Death Detection—Nuclear Double Staining

The cells were seeded on an 8-well chamber slide at a density of 5 × 10^4^ cells/mL. The next day, the culture medium was removed and the cells were exposed to BVR at concentrations of 4.31 µg/mL (LS180) or 46.06 µg/mL (HT-29). After 48 h of treatment, cell death was visualized using nuclear double-staining. The cancer cells were incubated with a fluorochrome mixture: propidium iodide (0.15 mg/mL) and Hoechst 33342 (0.24 mg/mL) for 5 min. Then, the stained cells were observed under a fluorescence microscope (Olympus BX51 System Microscope, Olympus Optical CO, Ltd., Tokyo, Japan). Cell images were captured using the CellFamily AnalySIS software (CellFamily AnalySIS software v 3.3) (Matrix Optics, Petaling Jaya, Malaysia).

### 4.9. Gene Expression Analysis

The cells were seeded in 6-well plates at a density of 5 × 10^4^ cells/mL. The next day, the culture medium was removed and the cells were exposed to BVR at concentrations equal to the IC_50_ values calculated on the basis of the results of the MTT assay performed on the cell lines. After 48 h of treatment, the cells were washed with ice-cold PBS and harvested. Then, the total RNA was extracted using a HighPure RNA Isolation Kit (Roche Diagnostics GmbH, Mannheim, Germany) according to the manufacturer’s instructions. The amount and purity of isolated nucleic acids were determined spectrophotometrically using NanoDrop (Thermo Scientific, Wilmington, DE, US). 

In total, 1 µg of total RNA was reverse transcripted using the SuperScript Vilo cDNA Synthesis kit according to the manufacturer’s recommendations. Subsequently, the cDNA was stored at −20 °C until later analysis. Before polymerase chain reaction (PCR), the cDNA was diluted 20 times and reactions were performed using SG onTaq qPCR Master Mix (Eurx, Gdańsk, Poland) in the QuantStudio 5 real-time PCR system (Applied Biosystems, Waltham, MA, USA) under the thermal cycling conditions given in the mix manual, for 40 amplification cycles with an annealing/elongation step at 60 °C for 1 min. A melt curve analysis was performed after each reaction plate. All samples were evaluated in triplicate in three technical repetitions (nine replicates in total in both the control and exposed groups). The expression of the following genes was examined: *BCL2* apoptosis regulator (*BCL2*), BCL2 like 1 (*BCL2L1*), BCL2 like 2 (*BCL2L2*), caspase 3 (*CASP3*), and caspase 9 (*CASP9*). Both the glyceraldehyde-3-phosphate dehydrogenase (*GAPDH)* and hypoxanthine phosphoribosyltransferase 1 (*HPRT1*) genes were used for expression normalization. All primers, except those previously used for *GAPDH* [109], were designed by the Primer-BLAST online tool [110]. A detailed characterization of these primers is presented in Supplementary Table S1. The specificity of the PCR products was evaluated in 2% agarose gel electrophoresis. Data were collected and analysed using the comparative Ct method (ΔΔCt method; QuantStudio Design and Analysis Software v1.5.2, Applied Biosystems, Waltham, MA, USA). The expression results are presented as a relative quantification (RQ).

### 4.10. Phytochemical Profiling and Quantification of Major Specialized Metabolites Using LC-UV-MS/MS Technique

A high-resolution MS analysis of the BVR extract was performed using a Bruker Impact II HD (Bruker, Billerica, MA, USA) quadrupole time-of-flight mass spectrometer (Q-TOF-MS) coupled with an Ultimate 3000 RS chromatographic system (Thermo Fischer Scientific, Wilmington, DE, US). The sample was chromatographed on a Cortecs T3 column (2.1 × 150 mm, 2.7 μm, Waters, Milford, MA, USA) equipped with a precolumn. A 25 min linear gradient (5→50%) of an acetonitrile–water mixture (both acidified with 0.1% formic acid) with a flow rate of 0.5 mL/min was applied. The column was kept at 40 °C. The injection volume was 2 µL. The MS operated in electrospray ionization and both polarity modes, with the following settings: mass scan range of 50–1200 *m/z*; capillary voltage of 4.0 kV (ESI+) or 3.0 kV (ESI–); nebuliser and drying gas (N_2_) of 2.0 bar and 10 L/min, respectively; and dry gas temperature of 220 °C. The MS/MS spectra were registered using a collision energy of 35 eV with stepping at 50% and 125% of CE. The acquired data were calibrated internally with sodium formate (10 mM solution in 50% 2-propanol) which was injected into the ion source before the sample analysis. The MS acquisition was accompanied by a charged aerosol detector (CAD, Corona Veo RS, Thermo) that collected data at a frequency of 10 Hz. The data were processed using the DataAnalysis 4.4 software (Bruker, Billerica, MA, USA). 

A quantitative analysis of the alkaloids and phenolic acids was carried out using a UPLC-PDA system (ACQUITY class, Waters, Milford, MA, USA). 

The chromatographic conditions were as follows: BEH C18 column (2.1 × 100 mm, 1.7 μm, Waters, Milford, MA, USA) maintained at 45 °C, a linear gradient of 28 min (5→40%) of a methanol–water mixture (both acidified with 0.1% formic acid), a flow rate of 0.25 mL/min, and a 2.5 µL injection volume. 

The alkaloids were detected based on UV 270 nm (3.6 nm resolution) and a calibration curve for berberine (y = 882.3 x − 287.85; R^2^ = 0.9995; linear range 0.5–30 µg/mL; LOD = 58.85 ng/mL; LOQ = 176.55 ng/mL). The quantitative results are expressed as milligrams of berberine equivalents (BE) per gram of extract (BVR).

A quantitative analysis of the content of phenolic acid derivatives was performed using the system and chromatographic conditions as previously described, although UV 320 nm detection and a calibration curve for ferulic acid (y = 2716.55x − 67.37; R^2^ = 0.9999; range 0.5–19.8 µg/mL; LOD = 18.65 ng/mL; LOQ = 55.95 ng/mL) were used. The quantitative results are expressed as milligrams of ferulic acid equivalents (FAE) per gram of extract (BVR). 

Data were collected and processed using the MassLynx 4.1 software (Waters, Milford, MA, USA).

### 4.11. Statistical Analysis

Quantitative values with normal distribution are presented as mean ± standard deviations (SD) or standard errors (SEM), otherwise, medians (lower–upper quartiles) were used. Depending on the distribution, assessed by the Shapiro–Wilk W test, differences between the two independent groups were compared by Student’s *t*-test, otherwise, the Mann–Whitney U test was used. A comparison between more than two groups was performed using one-way ANOVA, followed by Dunnett’s comparison test. A *p*-value less than 0.05 was considered to be statistically significant. Data were analysed using Statistica 13.0 (Statsoft Inc., Tulsa, OK, USA) and Microsoft Excel software (Microsoft Excel 2019). The IC_50_ value (concentration leading to 50% inhibition of cell viability compared to the control) was calculated using GraphPad PRISM (GraphPad Prism 5.0.).

## 5. Conclusions

Despite quite intensive research on the activity of the metabolites isolated from *B. vulgaris* L. against colon cancer, there is still very little research on the activity of extracts from this plant. Analyzing the literature data concerning the activity of other metabolites also unidentified in this study, it can be concluded that anticancer activity against colorectal cancer should be one of the main and most justified research directions concerning BVR. However, data on this topic are very lacking in the literature. The present study showed that the root of *B. vulgaris* L. is a raw material with a high chemopreventive potential to prevent the development of colorectal cancer. The extract is noteworthy because its action is closely related to impacts on processes related to the occurrence of non-modifiable risk factors for colorectal cancer (inflammatory bowel disease, and polyps), which, combined with the modification of modifiable factors (lifestyle, diet, and obesity), can significantly reduce the overall risk of developing colon cancer. Due to its antioxidant, anti-inflammatory, and antiproliferative effects, it can be treated as a comprehensive chemopreventive agent in patients at risk of developing colorectal cancer. It especially concerns those suffering from inflammatory bowel disease or polyps, which are non-modifiable risk factors for colorectal cancer, characterized by inflammation, increased oxidative stress, and impaired apoptosis. BVR is cytotoxic to colorectal cancer cells, but has less cytotoxicity to normal cells. The extract increases the expression of genes related to the apoptosis of colorectal cancer cells, inhibiting tumor proliferation and development. 

The raw material of the root of *B. vulgaris* L. is rich in alkaloids with proven anticancer effectiveness. The quantitative content of these compounds determined in this study allows us to conclude that they act in BVR at much lower doses than in the case of the independent action of a single compound, which can provide an opportunity to reduce the dose of the drug by using BVR. Synergistic action or the influence of undetermined metabolites are the probable reason for BVR activity. However as shown in this study, BVR has an appropriate quantity of pharmacologically active alkaloids responsible for its pro-apoptotic activity. Nonetheless, further research to better understand the chemical composition of BVR seems to be very important. Our finding may be considered as an initial study, and further analysis should be performed in order to improve or replicate our results. This research should be carried out on a larger number of biological replicates to minimize the dispersion of the results obtained. On the other hand, this study may be an incentive for other scientists to become interested in this important topic from a clinical point of view.

An additional advantage is the safety of using the raw material due to the centuries-old tradition of its use, which is why it can be treated as a dietary supplement or a traditional medicine—the French Pharmacopoeia contains a monograph on the bark of the root of *B. vulgaris* L. 

## Figures and Tables

**Table 1:** Antioxidant and anti-lipoxygenase activity of BVR. Antiradical effects were determined using ABTS•+, DPPH•, and ORAC assays, and the results are expressed as Trolox equivalents (mg Trolox/g of dry extract). In the LOX test, the results are expressed as % of lipoxygenase inhibition. Data are presented as means ± standard deviations of triplicate measurements.

ABTS•+ [mgTE/g]	ORAC [mgTE/g]	DPPH• [mgTE/g]	LOX Inhibition [%]
122.92 ± 0.01	220.29 ± 0.02	63.93 ± 0.01	62.60 ± 0.87

ABTS•+: 2,2′-azinobis-3-ethylbenzthiazoline-6-sulfonic acid; ORAC: Oxygen Radical Absorbance Capacity; DPPH•: 2,2-diphenyl-1-picrylhydrazyl radical; LOX: lipoxygenase.

**Table 2:** The IC_50_ values of BVR and berberine, palmatine, and berbamine estimated using the MTT assay. IC_50_ data are presented as means ± standard deviations of min. five measurements.

	CCD841 CoN	LS180	HT-29
	µg/mL	µg/mL	µg/mL
BVR	50.21 ± 1.22	4.31 ± 1.19	46.06 ± 1.11
Berberine	254.8 ± 2.05	0.45 ± 1.25	15.92 ± 1.08
Palmatine	179.8 ± 2.24	12.92 ± 1.10	29.34 ± 1.08
Berbamine	13.63 ± 1.02	37.62 ± 1.02	8.77 ± 1.07

**Table 3:** Expression of genes associated with apoptosis in LS180 and HT29 cells exposed to IC_50_ levels of BVR (4.31 and 46.06 µg/mL for LS180 and HT-29 cells, respectively). Values are expressed as means RQ ± standard deviations or medians [IQRs].

Gene	LS180			HT29		
	Control	Exposed	Change of Expression (%)	Control	Exposed	Change of Expression (%)
*CASP3*	0.46 [0.38–0.51]	1.14 [1.09–1.17]	247.83↑	0.57 ± 0.1	1.01 ± 0.11	177.19↑
*CASP9*	0.48 [0.44–0.6]	1.17 [1.01–1.31]	243.75↑	1.69 ± 0.25	2.67 ± 0.38	157.99↑
*BCL2*	1.23 ± 0.24	0.64 ± 0.12	52.03↓	0.12 [0.12–0.16]	0.03 [0.02–0.04]	25↓
*BCL2L1*	0.42 [0.39–0.57]	1.77 [1.44–1.86]	421.43↑	5.77 ± 1.12	7.32 ± 1.32	126.86↑
*BCL2L2*	0.55 [0.42–0.58]	1.32 [1.28–1.43]	240↑	0.93 [0.68–1.15	2.66 [2.55–2.8]	286.02↑

**Table 4:** Phytochemicals detected and quantified in BVR based on UHPLC-UV-CAD-MS/MS analysis. Compound numbers correspond to Figure 5.

No	RT (min)	Formula	Error (ppm)	Measured *m/z*	MS/MS Fragments	CAD Area (%)	Identity	Content [mg eq/g of Dry BVR] ± SD
1	0.81	-	0.0	341.1089 ^a^	179.0580, 119.0366	54.62	-	ui ^c^
2	7.28	C_16_H_19_O_9_	−0.4	355.1036 ^a^	193.0514, 78.0270, 134.0379	1.81	Feruloyl-hexoside	7.10 ^d^ ± 0.03
3	8.67	C_17_H_22_O_10_	−1.3	385.1145 ^a^	223.0624, 79.0699, 164.0471	0.22	Sinapoyl-hexoside	0.66 ^d^ ± 0.00
4	11.46	C_20_H_24_NO_4_	1.1	342.1695 ^b^	265.0854, 97.1117, 282.0882, 65.0694	1.89	Magnoflorine *	11.63 ^e^ ± 0.12
5	12.69	C_19_H_24_NO_3_	0.4	314.1749 ^b^	269.1169, 209.0957, 237.0907, 165.0697	0.64	Unidentified alkaloid	<LLOQ
6	14.06	C_36_H_39_N_2_O_6_	0.6	595.2799 ^b^	595.2798, 564.2381, 552.2376, 367.1648	6.04	Aromoline	7.11 ^e^ ± 0.22
7	17.16	C_37_H_40_N_2_O_6_	0.9	609.2954 ^b^	609.2955, 578.2539, 566.2538, 381.1804	2.46	Berbamine *	3.19 ^e^ ± 0.04
8	18.61	C_37_H_40_N_2_O_6_	0.4	609.2957 ^b^	609.2955, 381.1807, 174.0913, 578.2532	3.85	Oxycanthine	5.48 ^e^ ± 0.10
9	20.27	C_20_H_20_NO_4_	−0.2	338.1381 ^b^	322.1077, 308.0919, 294.1126, 236.0709	0.44	Columbamine	3.32 ^e^ ± 0.08
10	20.53	C_20_H_20_NO_4_	−0.5	338.1389 ^b^	322.1079,308.0920, 294.1128, 236.0708	4.4	Jatrorrhizine *	23.32 ^e^ ± 0.11
11	21.35	C_38_H_43_N_2_O_6_	0.9	623.3110 ^b^	623.3108, 381.1810, 174.0912, 592.2681	0.22	Rodiasine	<LLOQ
12	22.73	C_20_H_18_NO_4_	0.3	336.1229 ^b^	320.0916, 292.0966, 278.0809, 306.0760	19.34	Berberine *	70.27 ± 0.48
13	22.97	C_21_H_22_NO_4_	1.4	352.1537 ^b^	336.1226, 278.0807, 322.1073, 308.1278	4.07	Palmatine *	7.57 ^e^ ± 0.30

^a^ adduct [M-H]^–^, ^b^ adduct [M+H]^+^, ^c^ ui—unidetyfied, ^d^ ferulic acid equivalents (FAE), ^e^ berberine equivalents (BE) in mg BE per g BVR [mg BE/g BVR], *—the identity of metabolite was confirmed with the standards.

**Table 5:** Biological activity of BVR metabolite and the prospective of BVR further investigation.

Metabolite of the Root of *B. vulgaris* L.	Biological Activity of BVR Metabolite and Prospective of Further Investigation	References	Literature Confirmation of *B. vulgaris* L. Extract Activity	References
Bersavine Berberine Berbanine Berbamine	Antileukemic	[36,61,62,63,64,65,66]	Yes	[66,67]
Bersavine Berbamine Berberine Magnoflorine	Cervix cancer	[59,61,62,68]	No	-
Jatrorrhizine Berberine Magnoflorine	Liver cancer	[39,46,59,69,70,71,72]	Yes	[73]
Jatrorrhizine Berberine Bersavine Berbamine Magnoflorine Palmatine N-(p-trans-coumaroyl)tyramine cannabisin G (±)-lyoniresinol	Breast cancer	[46,60,61,62,69,74,75,76]	Yes	[21,77]
Berberine Jatrorrhizine Palmatine Columbamine Berbamine Bersavine	Colon cancer	[38,41,42,43,44,47,53,53,57,61,62,78]	No	-
Magnoflorine Berberine	Gastric cancer	[58,79]	No	-
Palmatine Berberine Jatrorrhizine	Skin cancer	[51,54,55,80]	No	-
Berberine, Columbamine, Isocorydine, Oxyberberine	Prostate cancer	[81]	No	-
Bersavine Berbamine Berberine	Lung cancer	[79,81,82]	No	-
(+)-chenabinol Berkristine Verfilline Bersavine Muraricine Berbostrejdine Aromoline Berlambine Obamegine Berberine Jathrrorhizine	Alzheimer disease	[48,83,84,85,86,87,88,89,90,91]	No	-
Berberine Magnoflorine Palmatine	Anti-inflammatory	[11,12,56,92]	Yes	[13,15,16,17,24,30,93]
Berberine Palmatine Jathrrorhizine	Anti-diabetic and Metabolic syndrome	[50,81,92,94]	Yes	[10,95,96,97,98,99,100,101]

## Data Availability

Data is contained within the article and supplementary material.

## Supplementary Materials

The following supporting information can be downloaded at: https://www.mdpi.com/article/10.3390/ijms25094786/s1.

## References

[1] World Cancer Research Fund International. https://www.wcrf-uk.org/.

[2] Labianca R., Nordlinger B., Beretta G.D., Mosconi S., Mandalà M., Cervantes A., Arnold D. (2013). Early colon cancer: ESMO Clinical Practice Guidelines for diagnosis, treatment and follow-up. Ann. Oncol..

[3] World Health Organisation. https://www.who.int/news-room/fact-sheets/detail/cancer..

[4] Simon K. (2016). Colorectal cancer development and advances in screening. Clin. Interv. Aging.

[5] Bardelčíková A., Šoltys J., Mojžiš J. (2023). Oxidative Stress, Inflammation and Colorectal Cancer: An Overview. Antioxidants.

[6] George B.P., Chandran R., Abrahamse H. (2021). Role of phytochemicals in cancer chemoprevention: Insights. Antioxidants.

[7] Maresso K.C., Tsai K.Y., Brown P.H., Szabo E., Lippman S., Hawk E.T. (2015). Molecular cancer prevention: Current status and future directions. CA A Cancer J. Clin..

[8] Yang D., Liu Y., Yong P.E., Qian Z., Xiao P. (2011). New collection of crude drugs in Chinese pharmacopoeia 2010 II. Sankezhen (*Berberis* spp.). Chin. Herb. Med..

[9] Henriette’s Herbal Homepage. https://www.henriettes-herb.com.

[10] Imanshahidi M., Hosseinzadeh H. (2008). Pharmacological and therapeutic effects of *Berberis vulgaris* and its active constituent, berberine. Phytother. Res..

[11] Li Y., Wang P., Chai M.J., Yang F., Li H.S., Zhao J., Wang H., Lu D.D. (2016). Effects of berberine on serum inflammatory factors and carotid atherosclerotic plaques in patients with acute cerebral ischemic stroke. China J. Chin. Mater. Medica.

[12] Meng S., Wang L.S., Huang Z.Q., Zhou Q., Sun Y.G., Cao J.T., Li Y.G., Wang C.Q. (2012). Berberine ameliorates inflammation in patients with acute coronary syndrome following percutaneous coronary intervention. Clin. Exp. Pharmacol. Physiol..

[13] Kiasalari Z., Khalili M., Ahmadi P. (2011). Effect of alcoholic extract of *Berberis vulgaris* fruit on acute and chronic inflammation in male rats. J. Babol Univ. Med. Sci..

[14] Minaiyan M., Ghannadi A., Mahzouni P., Jaffari-Shirazi E. (2011). Comparative study of *Berberis vulgaris* fruit extract and berberine chloride effects on acetic acid-induced colitis in rats. Iran. J. Pharm. Res..

[15] Majeed W., Aslam B., Javed I., Khaliq T., Muhammad F., Ali A., Raza A. (2015). Histopathological evaluation of gastro protective effect of *Berberis vulgaris* (Zereshk) seeds against aspirin induced ulcer in albino mice. Pak. J. Pharm. Sci..

[16] Mohebali S., Nasri S., Kamalinejhad M., Noori A.S. (2011). Antinociceptive & anti-inflammatory effects of *Berberis vulgaris* L. root’s hydroalcoholic extract and determination of it’s possible antinociceptive mechanism in male mice. J. Paramed. Sci. (JPS).

[17] Ivanovska N., Philipov S. (1996). Study on the anti-inflammatory action of *Berberis vulgaris* root extract, alkaloid fractions and pure alkaloids. Int. J. Immunopharmacol..

[18] ClinicalTrials.gov. https://clinicaltrials.gov/.

[19] Abd El-Wahab A.E., Ghareeb D.A., Sarhan E.E.M., Abu-Serie M.M., el Demellawy M.A. (2013). In vitro biological assessment of *Berberis vulgaris* and its active constituent, berberine: Antioxidants, anti-acetylcholinesterase, anti-diabetic and anticancer effects. BMC Complement. Altern. Med..

[20] Gird C.E., Ligiaelena D.U., Costea T., Nencu I., Popescu M., Balaci T., Olaru O. (2017). Research regarding obtaining herbal extracts with antitumor activity. note ii. phytochemical analysis, antioxidant activity and cytotoxic effects of *Chelidonium majus* L., Medicago sativa L. and *Berberis vulgaris* L. dry extracts. Medicago sativa L. and Berberis vulgaris L. dry extracts. Farmacia.

[21] Ghafourian E., Sadeghifard N., Pakzad I., Valizadeh N., Maleki A., Jafari F., Ghiasvand N., Abdi J., Shokoohinia Y., Ghafourian S. (2017). Ethanolic extract of *Berberis vulgaris* fruits inhibits the proliferation of MCF-7 breast cancer cell line through induction of apoptosis. Former. Curr. Drug Targets-Infect. Disord..

[22] El Khalki L., Tilaoui M., Jaafari A., Ait Mouse H., Zyad A. (2018). Studies on the Dual Cytotoxicity and Antioxidant Properties of *Berberis vulgaris* Extracts and Its Main Constituent Berberine. Adv. Pharmacol. Sci..

[23] Lin C.C., Ng L.T., Hsu F.F., Shieh D.E., Chiang L.C. (2004). Cytotoxic effects of *Coptis chinensis* and *Epimedium sagittatum* extracts and their major constituents (berberine, coptisine and icariin) on hepatoma and leukaemia cell growth. Clin. Exp. Pharmacol. Physiol..

[24] Och A., Olech M., Bąk K., Kanak S., Cwener A., Cieśla M., Nowak R. (2023). Evaluation of the antioxidant and anti-lipoxygenase activity of *Berberis vulgaris* L. leaves, fruits, and stem and their LC MS/MS polyphenolic profile. Antioxidants.

[25] Gorizpa M., Bahmanyar F., Mirmoghtadaie L., Shafaei F. (2022). Evaluation of Antioxidant and Antimicrobial Properties of Root and Stem Bark Extracts of Three Species of Barberry in Bread. Res. Innov. Food Sci. Technol..

[26] Luo A., Fan Y. (2011). Antioxidant activities of berberine hydrochloride. J. Med. Plants Res..

[27] Chaves S.K., Afzal M.I., Islam M.T., Hameed A., Da Mata A.M., da Silva Araújo L., Ali S.W., Rolim H.M., De Medeiros M.D., Costa E.V. (2020). Palmatine antioxidant and anti-acetylcholinesterase activities: A pre-clinical assessment. Cell. Mol. Biol..

[28] Villinski J.R., Dumas E.R., Chai H.B., Pezzuto J.M., Angerhofer C.K., Gafner S. (2003). Antibacterial activity and alkaloid content of *Berberis thunbergii*, *Berberis vulgaris* and Hydrastis canadensis. Pharm. Biol..

[29] Xu T., Kuang T., Du H., Li Q., Feng T., Zhang Y., Fan G. (2020). Magnoflorine: A review of its pharmacology, pharmacokinetics and toxicity. Pharmacol. Res..

[30] Kalmarzi R.N., Naleini S.N., Ashtary-Larky D., Peluso I., Jouybari L., Rafi A., Ghorat F., Heidari N., Sharifian F., Mardaneh J. (2019). Anti-inflammatory and immunomodulatory effects of barberry (*Berberis vulgaris*) and its main compounds. Oxidative Med. Cell. Longev..

[31] Sarraf M., Beig Babaei A., Naji-Tabasi S. (2019). Investigating functional properties of barberry species: An overview. J. Sci. Food Agric..

[32] Guo S., Jiang K., Wu H., Yang C., Zhao G., Deng G. (2018). Magnoflorine ameliorates lipopolysaccharide-induced acute lung injury via suppressing NF-κB and MAPK activation. Front. Pharmacol..

[33] Mokhber-Dezfuli N., Saeidnia S., Gohari A., Kurepaz-Mahmoodabadi M. (2014). Phytochemistry and pharmacology of berberis species. Pharmacogn. Rev..

[34] Bhardwaj D., Kaushik N. (2012). Phytochemical and pharmacological studies in genus *Berberis*. Phytochem. Rev..

[35] Li Q., Zhao H., Chen W., Huang P. (2023). Berberine induces apoptosis and arrests the cell cycle in multiple cancer cell lines. Arch. Med. Sci. AMS.

[36] Och A., Zalewski D., Komsta Ł., Kołodziej P., Kocki J., Bogucka-Kocka A. (2019). Cytotoxic and proapoptotic activity of sanguinarine, berberine, and extracts of chelidonium majus L. and berberis thunbergii DC. Toward hematopoietic cancer cell lines. Toxins.

[37] Bala M., Pratap K., Verma P.K., Singh B., Padwad Y. (2015). Validation of ethnomedicinal potential of Tinospora cordifolia for anticancer and immunomodulatory activities and quantification of bioactive molecules by HPTLC. J. Ethnopharmacol..

[38] Mou L., Liang B., Liu G., Jiang J., Liu J., Zhou B., Huang J., Zang N., Liao Y., Ye L. (2019). Berbamine exerts anticancer effects on human colon cancer cells via induction of autophagy and apoptosis.; inhibition of cell migration and MEK/ERK signalling pathway. J. BUON.

[39] Zhang L.L., Li-Na M.A., Dan Y.A., Zhang C.E., Dan G.A., Xiong Y., Sheng F.Y., Xiao-Ping D.O., Xiao-He X.I. (2014). Dynamic monitoring of the cytotoxic effects of protoberberine alkaloids from Rhizoma Coptidis on HepG2 cells using the xCELLigence system. Chin. J. Nat. Med..

[40] Kalaiarasi A., Anusha C., Sankar R., Rajasekaran S., John Marshal J., Muthusamy K., Ravikumar V. (2016). Plant isoquinoline alkaloid berberine exhibits chromatin remodeling by modulation of histone deacetylase to induce growth arrest and apoptosis in the A549 cell line. J. Agric. Food Chem..

[41] Su Y.H., Tang W.C., Cheng Y.W., Sia P., Huang C.C., Lee Y.C., Jiang H.Y., Wu M.H., Lai I.L., Lee J.W. (2015). Targeting of multiple oncogenic signaling pathways by Hsp90 inhibitor alone or in combination with berberine for treatment of colorectal cancer. Biochim. Et Biophys. Acta (BBA)-Mol. Cell Res..

[42] Wu K., Yang Q., Mu Y., Zhou L., Liu Y., Zhou Q., He B. (2012). Berberine inhibits the proliferation of colon cancer cells by inactivating Wnt/β-catenin signaling. Int. J. Oncol..

[43] Chidambara Murthy K.N., Jayaprakasha G.K., Patil B.S. (2012). The natural alkaloid berberine targets multiple pathways to induce cell death in cultured human colon cancer cells. Eur. J. Pharmacol..

[44] Dai W., Mu L., Cui Y., Li Y., Chen P., Xie H., Wang X. (2019). Berberine promotes apoptosis of colorectal cancer via regulation of the long non-coding RNA (lncRNA) cancer susceptibility candidate 2 (CASC2)/AU-binding factor 1 (AUF1)/B-cell CLL/lymphoma 2 (Bcl-2) axis. Med. Sci. Monit. Int. Med. J. Exp. Clin. Res..

[45] Vuddanda P.R., Chakraborty S., Singh S. (2010). Berberine: A potential phytochemical with multispectrum therapeutic activities. Expert Opin. Investig. Drugs.

[46] Sun Y., Gao X., Wu P., Wink M., Li J., Dian L., Liang Z. (2019). Jatrorrhizine inhibits mammary carcinoma cells by targeting TNIK mediated Wnt/β-catenin signalling and epithelial-mesenchymal transition (EMT). Phytomedicine.

[47] Wang P., Gao X.Y., Yang S.Q., Sun Z.X., Dian L.L., Qasim M., Phyo A.T., Liang Z.S., Sun Y.F. (2019). Jatrorrhizine inhibits colorectal carcinoma proliferation and metastasis through Wnt/β-catenin signaling pathway and epithelial–mesenchymal transition. Drug Des. Dev. Ther..

[48] Luo T., Zhang H., Zhang W.W., Huang J.T., Song E.L., Chen S.G., He F., Xu J., Wang H.Q. (2011). Neuroprotective effect of Jatrorrhizine on hydrogen peroxide-induced cell injury and its potential mechanisms in PC12 cells. Neurosci. Lett..

[49] Kumari S., Kaladhar D.S., Solmon K.S., Malla R.R., Kishore G. (2013). Anti-proliferative and metastatic protease inhibitory activities of protoberberines: An in silico and in vitro approaches. Process Biochem..

[50] Rolle J., Asante D.O., Kok-Fong L.L., Boucetta H., Seidu T.A., Tai L.L.K., Alolga R.N. (2021). Jatrorrhizine: A review of its pharmacological effects. J. Pharm. Pharmacol..

[51] Ali D., Ali H. (2014). Assessment of DNA damage and cytotoxicity of palmatine on human skin epithelial carcinoma cells. Toxicol. Environ. Chem..

[52] Zhang Y.X., Zhang X.F., Tang Y.L., Xiang J.F., Tian M.Y. (2011). Studies of the interactions between three protoberberine alkaloids and Bcl-2 by fluorescence spectroscopy. Acta Chim. Sin..

[53] Wu J., Xiao O., Zhang N., Xue C., Leung A.W., Zhang H., Xu C., Tang Q. (2016). Photodynamic action of palmatine hydrochlo-ride on colon adenocarcinoma HT-29 cells. Photodiagnosis Photodyn. Ther..

[54] Inbaraj J.J., Kukielczak B.M., Bilski P., He Y.Y., Sik R.H., Chignell C.F. (2006). Photochemistry and photocytotoxicity of alkaloids from Goldenseal (*Hydrastis canadensis* L.). 2. Palmatine, hydrastine, canadine, and hydrastinine. Chem. Res. Toxicol..

[55] Hirakawa K., Kawanishi S., Hirano T. (2005). The mechanism of guanine specific photooxidation in the presence of berberine and palmatine: Activation of photosensitized singlet oxygen generation through DNA-binding interaction. Chem. Res. Toxicol..

[56] Ma W.K., Li H., Dong C.L., He X., Guo C.R., Zhang C.F., Yu C.H., Wang C.Z., Yuan C.S. (2016). Palmatine from Mahonia bealei attenuates gut tumorigenesis in ApcMin/+ mice via inhibition of inflammatory cytokines. Mol. Med. Rep..

[57] Lei C., Yao Y., Shen B., Liu J., Pan Q., Liu N., Li L., Huang J., Long Z., Shao L. (2019). Columbamine suppresses the proliferation and malignization of colon cancer cells via abolishing Wnt/β-catenin signaling pathway. Cancer Manag. Res..

[58] Sun X.L., Zhang X.W., Zhai H.J., Zhang D., Ma S.Y. (2020). Magnoflorine inhibits human gastric cancer progression by inducing autophagy, apoptosis and cell cycle arrest by JNK activation regulated by ROS. Biomed. Pharmacother..

[59] Mohamed S.M., Hassan E.M., Ibrahim N.A. (2010). Cytotoxic and antiviral activities of aporphine alkaloids of *Magnolia grandiflora* L.. Nat. Prod. Res..

[60] Bai L., Zhang H., Liu Q., Zhao Y., Cui X., Guo S., Zhang L., Ho C.T., Bai N. (2016). Chemical characterization of the main bioactive constituents from fruits of *Ziziphus jujuba*. Food Funct..

[61] Koutova D., Kulhava M., Havelek R., Majorosova M., Královec K., Habartova K., Hošťálková A., Opletal L., Cahlikova L., Řezáčová M. (2020). Bersavine: A Novel Bisbenzylisoquinoline Alkaloid with Cytotoxic, Antiproliferative and Apoptosis-Inducing Effects on Human Leukemic Cells. Molecules.

[62] Habartová K., Havelek R., Seifrtova M., Hostalkova A., Cahlikova L., Rezacova M. (2017). A new isoquinoline alkaloid bersavine as a possible anticancer agent. Ann. Oncol..

[63] Hošt’álková A., Novák Z., Pour M., Jirošová A., Opletal L., Kuneš J., Cahlíková L. (2013). Berbanine: A new isoquinoline-isoquinolone alkaloid from *Berberis vulgaris* (Berberidaceae). Nat. Prod. Commun..

[64] Liang Y., Qiu X., Xu R.Z., Zhao X.Y. (2011). Berbamine inhibits proliferation and induces apoptosis of KU812 cells by increasing Smad3 activity. J. Zhejiang Univ. Sci. B.

[65] Liang Y., Xu R.Z., Zhang L., Zhao X.Y. (2009). Berbamine, a novel nuclear factor κB inhibitor, inhibits growth and induces apoptosis in human myeloma cells. Acta Pharmacol. Sin..

[66] Hussain S.F. (2018). Secobisbenzylisoquinoline alkaloids-chemistry and pharmacology: A review. Baqai J. Health Sci..

[67] Saedi T.A., Ghafourian S., Jafarlou M., Sabariah M.N., Ismail P., Eusni R.M., Othman F. (2015). *Berberis vulgaris* fruit crude extract as a novel anti-leukaemic agent. J. Biol. Regul. Homeost. Agents.

[68] Liu L., Sun L., Zheng J., Cui L. (2021). Berberine modulates Keratin 17 to inhibit cervical cancer cell viability and metastasis. J. Recept. Signal Transduct..

[69] Zhu Y., Xie N., Chai Y., Nie Y., Liu K., Liu Y., Yang Y., Su J., Zhang C. (2022). Apoptosis induction, a sharp edge of berberine to exert anti-cancer effects, focus on breast, lung, and liver cancer. Front. Pharmacol..

[70] Wang N., Feng Y., Zhu M., Tsang C.M., Man K., Tong Y., Tsao S.W. (2010). Berberine induces autophagic cell death and mitochondrial apoptosis in liver cancer cells: The cellular mechanism. J. Cell. Biochem..

[71] Zhang P., Wang Q., Lin Z., Yang P., Dou K., Zhang R. (2019). Berberine inhibits growth of liver cancer cells by suppressing glutamine uptake. OncoTargets Ther..

[72] Liu Y., Nwafor E.O., Li Z., Wang J., Feng X., Li H., Jia B., Ma H., He J., Pi J. (2022). Effects of Berberine on Liver Cancer. Nat. Prod. Commun..

[73] Hanachi P., Othman F., Motaleb G.R. (2008). Effect of *Berberis vulgaris* aqueous extract on the apoptosis, sodium and potassium in hepatocarcinogenic rats. Iran. J. Basic Med. Sci..

[74] Wu J., Xiao Q., Zhang N., Xue C., Leung A.W., Zhang H., Tang Q.J., Xu C. (2016). Palmatine hydrochloride mediated photodynamic inactivation of breast cancer MCF-7 cells: Effectiveness and mechanism of action. Photodiagnosis Photodyn. Ther..

[75] Kaboli P.J., Rahmat A., Ismail P., Ling K.H. (2014). Targets and mechanisms of berberine, a natural drug with potential to treat cancer with special focus on breast cancer. Eur. J. Pharmacol..

[76] Tomosaka H., Chin Y.W., Salim A.A., Keller W.J., Chai H., Kinghorn A.D. (2008). Antioxidant and cytoprotective compounds from *Berberis vulgaris* (barberry). Phytother. Res..

[77] Hoshyar R., Mahboob Z., Zarban A. (2016). The antioxidant and chemical properties of *Berberis vulgaris* and its cytotoxic effect on human breast carcinoma cells. Cytotechnology.

[78] Wang L., Liu L., Shi Y., Cao H., Chaturvedi R., Calcutt M.W. (2012). Berberine induces caspase-independent cell death in colon tumor cells through activation of apoptosis-inducing factor. PLoS ONE.

[79] Liu B.S., Liu K., Wang J., Shi Y.M. (2023). Anticancer Potential of Nature-Derived Isoquinoline Alkaloids (A Review). Russ. J. Gen. Chem..

[80] Liu R., Cao Z., Pan Y., Zhang G., Yang P., Guo P., Zhou Q. (2013). Jatrorrhizine hydrochloride inhibits the proliferation and neovascularization of C8161 metastatic melanoma cells. Anti-Cancer Drugs.

[81] Sindhoor S.M., Naveen N.R., Rao G.K., Gopan G., Chopra H., Park M.N., Alshahrani M.M., Jose J., Emran T.B., Kim B. (2022). A spotlight on alkaloid nanoformulations for the treatment of lung cancer. Front. Oncol..

[82] Alam M., Abbas K., Raza M.T., Yahya H., Saifi M.F., Kamal S. (2024). Evaluation of *Berberis vulgaris* Phytochemicals for Targeting PIM1 Kinase in Prostate Cancer: An In silico Approach. Int. Res. J. Oncol..

[83] Hostalkova A., Marikova J., Opletal L., Korabecny J., Hulcova D., Kunes J., Novakova L., Perez D.I., Jun D., Kucera T. (2019). Isoquinoline Alkaloids from *Berberis vulgaris* as Potential Lead Compounds for the Treatment of Alzheimer’s Disease. J. Nat. Prod..

[84] Hošťálková A., Novák Z., Hrabinová M., Štěpánková Š., Peréz D., Kuneš J., Cahlíková L., Opletal L. (2015). Alkaloids from *Berberis vulgaris* and their biological activity connected to Alzheimer’s disease. Planta Medica.

[85] Novák Z., Hošt’álková A., Opletal L., Nováková L., Hrabinová M., Kuneš J., Cahliková L. (2015). (+)-chenabinol (revised NMR data) and two new alkaloids from *Berberis vulgaris* and their biological activity. Nat. Prod. Commun..

[86] Zeng X.H., Zeng X.J., Li Y.Y. (2003). Efficacy and safety of berberine for congestive heart failure secondary to ischemic or idiopathic dilated cardiomyopathy. Am. J. Cardiol..

[87] Barrios V., Escobar C., Cicero A.F.G., Burke D., Fasching P., Banach M., Bruckert E. (2017). A nutraceutical approach (Armolipid Plus) to reduce total and LDL cholesterol in individuals with mild to moderate dyslipidemia: Review of the clinical evidence. Atheroscler. Suppl..

[88] Li X.Y., Zhao Z.X., Huang M., Feng R., He C.Y., Ma C., Luo S.-H., Fu J., Wen B.Y., Ren L. (2015). Effect of Berberine on promoting the excretion of cholesterol in high-fat diet-induced hyperlipidemic hamsters. J. Transl. Med..

[89] Brusq J.M., Ancellin N., Grondin P., Guillard R., Martin S., Saintillan Y., Issandou M. (2006). Inhibition of lipid synthesis through activation of AMP kinase: An additional mechanism for the hypolipidemic effects of berberine. J. Lipid Res..

[90] Wang L., Peng L., Wei G., Ge H. (2016). Therapeutic Effects of Berberine Capsule on Patients with Mild Hyperlipidemia. Zhongguo Zhong Xi Yi Jie He Za Zhi Zhongguo Zhongxiyi Jiehe Zazhi Chin. J. Integr. Tradit. West. Med..

[91] Luo T., Shen X.Y., Li S. (2017). The protective effect of jatrorrhizine against oxidative stress in primary rat cortical neurons. CNS Neurol. Disord. Drug Targets.

[92] Och A., Podgórski R., Nowak R. (2020). Biological activity of berberine—A summary update. Toxins.

[93] Nor N.M., Noor S.M., Tohit E.M., Othman F. (2020). Anti-inflammatory effect of *Berberis vulgaris* extract in vivo. Atherosclerosis.

[94] Och A., Och M., Nowak R., Podgórska D., Podgórski R. (2022). Berberine, a herbal metabolite in the metabolic syndrome: The risk factors, course, and consequences of the disease. Molecules.

[95] Tabeshpour J., Imenshahidi M., Hosseinzadeh H. (2017). A review of the effects of *Berberis vulgaris* and its major component, berberine, in metabolic syndrome. Iran. J. Basic Med. Sci..

[96] Och A., Nowak R. (2021). Barberry (Berberis vulgaris)—Traditional and Contemporary Use. Medicinal Plants: Domestication, Biotechnology and Regional Importance.

[97] Zilaee M., Kermany T., Tavalaee S., Salehi M., Ghayour-Mobarhan M., Ferns G.A.A. (2014). Barberry treatment reduces serum antiheat shock protein 27 and 60 antibody titres and high-sensitivity C-reactive protein in patients with metabolic syndrome: A double-blind, randomized placebo-controlled trial. Phytother. Res..

[98] Mohammadi A., Sahebkar A., Kermani T., Zhilaee M., Tavallaie S., Ghayour Mobarhan M. (2014). Barberry administration and pro-oxidant–antioxidant balance in patients with metabolic syndrome. Iran. Red Crescent Med. J..

[99] Ebrahimi-Mamaghani M., Arefhosseini S.R., Golzarand M., Aliasgarzadeh A. (2009). Long-term effects of processed *Berberis vulgaris* on some metabolic syndrome components. Iran. J. Endocrinol. Metab..

[100] Meliani N., Dib M.A., Allali H., Tabti B. (2011). Hypoglycaemic effect of *Berberis vulgaris* L. in normal and streptozotocin-induced diabetic rats. Asian Pac. J. Trop. Biomed..

[101] Safari Z., Farrokhzad A., Ghavami A., Fadel A., Hadi A., Rafiee S., Mokari-Yamchi A., Askari G. (2020). The Effect of Barberry (*Berberis vulgaris* L.) on Glycemic Indices: A Systematic Review and Meta-Analysis of Randomized Controlled Trials. Complement. Ther. Med..

[102] Kukula-Koch W., Koch W., Angelis A., Halabalaki M., Aligiannis N. (2016). Application of pH-zone refining hydrostatic countercurrent chromatography (hCCC) for the recovery of antioxidant phenolics and the isolation of alkaloids from Siberian barberry herb. Food Chem..

[103] Brand-Williams W., Cuvelier M.E., Berset C. (1995). Use of a Free Radical Method to Evaluate Antioxidant Activity. LWT Food Sci. Technol..

[104] Olech M., Łyko L. (2020). Nowak R, Influence of Accelerated Solvent Extraction Conditions on the LC-ESI-MS/MS Polyphenolic Profile, Triterpenoid Content, and Antioxidant and Anti-lipoxygenase Activity of Rhododendron luteum Sweet Leaves. Antioxidants.

[105] Re R., Pellegrini N., Proteggente A., Pannala A., Yang M., Rice-Evans C. (1999). Antioxidant activity applying an improved ABTS radical cation decolorization assay. Free Radic. Biol. Med..

[106] Pieczykolan A., Pietrzak W., Nowak R., Pielczyk J., Łamacz K. (2019). Optimization of Extraction Conditions for Determination of Tiliroside in Tilia L, Flowers Using an LC-ESI-MS/MS Method. J. Anal. Methods Chem..

[107] Huang D., Ou B., Hampsch-Woodill M., Flanagan J.A., Prior R.L. (2002). High-Throughput Assay of Oxygen Radical Absorbance Capacity (ORAC) Using a Multichannel Liquid Handling System Coupled with a Microplate Fluorescence Reader in 96-Well Format. J. Agric. Food Chem..

[108] Baraniak B., Szymanowska U. (2006). Lipooxygenase in food of plant origin. Żywność Nauka Technol. Jakość.

[109] Podgórski R., Cieśla M., Podgórska D., Bajorek W., Płonka A., Czarny W., Trybulski R., Król P. (2022). Plasma microRNA-320a as a Potential Biomarker of Physiological Changes during Training in Professional Volleyball Players. J. Clin. Med..

[110] Ye J., Coulouris G., Zaretskaya I., Cutcutache I., Rozen S., Madden T.L. (2012). Primer-BLAST: A tool to design target-specific primers for polymerase chain reaction. BMC Bioinform..

