# Supplementary material for: Berberis vulgaris L. Root Extract as a Multi-Target Chemopreventive Agent against Colon Cancer Causing Apoptosis in Human Colon Adenocarcinoma Cell Lines
Source: Int J Mol Sci. 2024 Apr 27;25(9):4786. doi: 10.3390/ijms25094786 (PMC11084310; doi:10.3390/ijms25094786)
Supplement: Supplementary file 1 [file ijms-25-04786-s001.zip › ijms-2949648-supplementary.pdf]

Table S1. Primers sequences used in gene expression analysis.

Gene name	Primer sense 5′→3′	Primer antisense 5′→3′	Final concentration in PCR [nM]
GAPDH	AGAAGGCTGGGGCTCATTTG	TGATGGCATGGACTGTGGTCAT	100
HPRT1	GCTCCGTTATGGCGACCC	GCAAGACGTTCAGTCCTGTCC	450
BCL2	CGCGACTCCTGATTCATTGG	GTCTACTTCCTCTGTGATGTTGT	300
BCL2L1	CGATGGAGGAGGAAGCAAGC	TCTGAAGGGAGAGAAAGAGATTCAA	300
BCL2L2	GGGGCTGGGCGGAGTT	CTCCACTTCTTCCAAGGGCA	450
CASP3	CAGTGGAGGCCGACTTCTTG	GCACAAAGCGACTGGATGAA	450
CASP9	TCCTACTCTACTTTCCCAGGTTT	CCCACTGCTCAAAGATGTCG	450
